# Reniform Nematodes from Various Geographic Origins Uniquely Influenced Cotton Development

**DOI:** 10.3390/plants15121904

**Published:** 2026-06-19

**Authors:** Sagar GC, Churamani Khanal

**Affiliations:** Department of Plant and Environmental Sciences, Clemson University, 105 Collings St., Clemson, SC 29634, USA; sagarg@g.clemson.edu

**Keywords:** cotton, development, management, nematode, physiology, variability

## Abstract

Greenhouse studies were conducted to assess the impact of reniform nematode (*Rotylenchulus reniformis*) isolates originating from nine states in the US cotton belt (TN, AL, MS, LA, TX, AR, FL, SC, and GA) on the development of cotton. Two cotton cultivars, DP 2141NR B3XF, marketed as reniform nematode resistant, and DP 2317 B3TXF, a susceptible control, were employed. Origin of the reniform nematode significantly influenced plant height, number of leaves, boll weight, chlorophyll content and plant vigor, but not photosynthesis and transpiration. While cotton plants inoculated with any of the isolates sustained negative developmental impacts in comparison with the uninoculated plants, the level of these impacts differed by the origin of the isolate. The isolates originating from the delta region (AR, MS and TN) had the most pronounced negative impacts on cotton. The development of plants inoculated with the FL, SC, TX, and LA isolates were moderately impacted while plants inoculated with the AL and the GA isolates showed the least amount of impact. Across all isolates and in comparison with the susceptible control, the resistant cultivar was taller, produced more leaves and bolls, and had superior vigor, chlorophyll content, photosynthesis and transpiration. As the ability of reniform nematode to impact cotton development can differ based on geography, results from this study implied a need for the development of niche-specific reniform nematode management methods in cotton.

## 1. Introduction

Cotton (*Gossypium hirsutum* L.) contributes about a quarter of the global fiber use [1]. The United States is one of the top cotton producers in the world and it represents 28% of global trade share [2]. The $4.6 billion dollar cotton industry in the United States faces threats from several pests including plant-parasitic nematodes [2,3]. These nematodes not only feed on plant roots but also provide avenues for other pathogens to enter the host through the wounds caused during feeding. Among several plant-parasitic nematodes, the reniform nematode (*Rotylenchulus reniformis* Linford and Oliveira) is one of the most damaging pathogens of cotton [4,5]. Despite the use of pre-plant fumigation and the availability of a few cultivars claimed by the seed industries as resistant to reniform nematode, the United States cotton industry lost 184 thousand bales of cotton to reniform nematode in 2024, which accounted for 22% of cotton lost to all diseases [6]. Unfortunately, abilities of currently available resistant cotton cultivars and breeding lines developed in the past to suppress reniform nematode damage have not been consistent when tested across multiple geographic locations [7,8,9,10].

One of the reasons behind inconsistency of resistant cotton genotypes is likely the inherent variability among nematode populations of different geographies. A few studies conducted in the past reported substantial variability in single egg mass-derived reniform nematode populations/isolates from within a state and between cotton producing states [11,12,13,14]. A recent study from our lab assessed the population-level response of reniform nematode isolates originating from nine cotton producing states in the United States and led to the same conclusion that inherent variability in reproduction and virulence exists among geographic populations of reniform nematode [15]. Additional data collected from that same study regarding the impact of reniform nematode isolates on crop growth parameters (plant height, number of leaves, plant vigor, and boll weight) as well as on crop physiology (chlorophyll content, photosynthesis and transpiration) are worth analyzing to make cotton researchers and other stakeholders aware of the nematode impact. While previous reports on the adverse impact of reniform nematode on cotton yield are available [16,17,18], to our knowledge, this study is the first to report the impact of reniform nematode on cotton development. This study also reports how resistant and susceptible cotton cultivars respond to reniform nematodes originating from geographically distinct populations.

## 2. Materials and Methods

The methodologies for the establishment of experiments were described in the recently published study from our lab [15] and are briefly described here. Field-derived or greenhouse-maintained populations of *R. reniformis* from Tennessee (TN), Alabama (AL), Mississippi (MS), Texas (TX), Louisiana (LA), Arkansas (AR), Florida (FL), South Carolina (SC), and Georgia (GA) were each increased by inoculating a two-week-old reniform nematode-susceptible cotton plant (cv DP1646 B2XF, Bayer, Whippany, NJ, USA). The soil used for population increase and subsequent experiment establishment was steam sterilized at 15 PSI for 45 min at 121 °C prior to pot filling. Nematode eggs were extracted from each isolate using a 10% commercial bleach solution containing 7.5% sodium hypochlorite according to a previously described method [19]. Inoculations were conducted on the same day as the egg extraction. Plants were watered daily and fertilized weekly using the recommended rate of Miracle Grow fertilizer (Scotts Miracle-Grow Company, Marysville, OH, USA) (N-P-K: 20-20-20). When needed, insect and spider management were conducted using Talstar^®^ Professional (Bifenthrin 7.9%, FMC Corporation, Philadelphia, PA, USA), Marathon (Imidacloprid 1%, OHP, Inc., Bluffton, SC, USA), or Sanmite^®^ (Pyridaben 75.0%, Gowan Company, Yuma, AZ, USA) following the manufacturer’s recommended rates. Four 1000 W metal halide bulbs hanging approximately 3 m above the table provided a 14 h photoperiod throughout the study period.

### 2.1. Establishment of Experiment

Two temporally spaced greenhouse experiments were established in a factorial treatment structure consisting of nine reniform nematode isolates (TN, AL, MS, TX, LA, AR, FL, SC, and GA) and two cotton cultivars (reniform nematode-resistant DP 2141NR B3XF and susceptible DP 2317 B3TXF). Non-inoculated resistant and susceptible cotton cultivars served as controls. Each experiment was established in a randomized complete block design with five replications. Cotton seeds were planted in 15 cm top-diameter plastic pots filled with 1.5 kg of steam sterilized soil. The soil contained 64% sand, 14% silt, 22% clay, 8 ppm of nitrate nitrogen, 134 lbs/acre of phosphorus, 161 lbs/acre of potassium, 1.5% organic matter, and had a cation exchange capacity of 3.1 meq/100 g. A week after seeding, plants were inoculated with an aqueous suspension of 10,000 reniform nematode eggs near the root zone of the cotton plant according to a previously described method [13]. The temperature and relative humidity of the greenhouse during the experimental period were maintained at 25 ± 5 °C and 82 ± 13% respectively. Each experiment was terminated 60 days after inoculation (DAI).

### 2.2. Data Collection

Measurements of plant heights and counting of fully expanded leaves were conducted at 30 and 60 DAI. The plant height was measured from the soil surface to the terminal bud [20]. Plant vigor was estimated through the Normalized Difference Vegetation Index (NDVI) of each plant biweekly (15, 30, 45, and 60 DAI) using a handheld crop sensor (GreenSeeker, Trimble, Westminster, CO, USA). White paper was placed on top of the soil, and the crop sensor was positioned one meter above the top of the plant prior to assessing plant vigor. Leaf chlorophyll content was estimated using a SPAD-502 (Minolta Camera Company, Osaka, Japan). Five SPAD (soil–plant analysis development) readings were collected in five random spots around the middle portion of the second fully expanded leaf from the top of each plant, and average values were recorded. To avoid potential damage to young leaves, SPAD readings were taken only at 15, 30, and 60 DAI. Photosynthesis and transpiration data were collected from the second fully expanded leaf from the top of each plant using a LI-6400XT portable photosynthesis system (LI-COR Inc., Lincoln, NE, USA) at 60 DAI. For consistency, NDVI, SPAD measurements, photosynthesis, and transpiration data were collected between 9 a.m. and 11 a.m. At the termination of each experiment, cotton bolls were harvested and kept in labeled paper bags, dried for a week at 65 °C in an incubator (VWR, Cornelius, OR, USA), and dry biomasses were recorded. Nematode reproduction data were collected at experiment termination by extracting eggs from each root system as described above. Roots after egg extraction were dried for a week at 65 °C in an incubator (VWR, Cornelius, OR, USA).

### 2.3. Data Analysis

Analysis of data was conducted using JMP Pro 18 (SAS Institute, Cary, NC, USA). Data were subjected to a two-way analysis of variance (ANOVA). Outliers were removed following residual analysis, and any non-normal data (NDVI at 45 DAI and SPAD at 60 DAI) were transformed using the square root function before subsequent analysis to meet the assumptions of ANOVA. Cotton cultivar and nematode isolate were considered as fixed effects, while replication and experiment were treated as random effects. Treatment means were compared using Student’s *t*-test or Tukey’s HSD test (*p* ≤ 0.05). Student’s *t*-test was used for the comparison of the main effect of cultivar on cotton development. Tukey’s HSD test was used for a *post* *hoc* mean comparison of the main as well as for the interactive effect of nematode isolate and cotton cultivar. Regression analysis was conducted using the Fit Y by X module to determine the relationship between nematode reproduction and crop parameters that were significantly impacted by interactions between nematode isolate and cotton cultivar. Nematode reproduction data were log transformed prior to analysis. Scatterplots and residual distributions were examined to assess the appropriateness of the linear regression model. Simple linear regression was performed using nematode reproduction data as the independent variable, and plant height, plant vigor and chlorophyll content as the dependent variables. The regression equations, coefficients of determination (R^2^) and *p*-values were obtained from the fitted models and visualized using the Graph Builder module. When transformed, the letters of significance presented in tables and figures came from the transformation analysis while the treatment means presented were untransformed values.

## 3. Results

### 3.1. Impact on Plant Height

**Figure 1:**
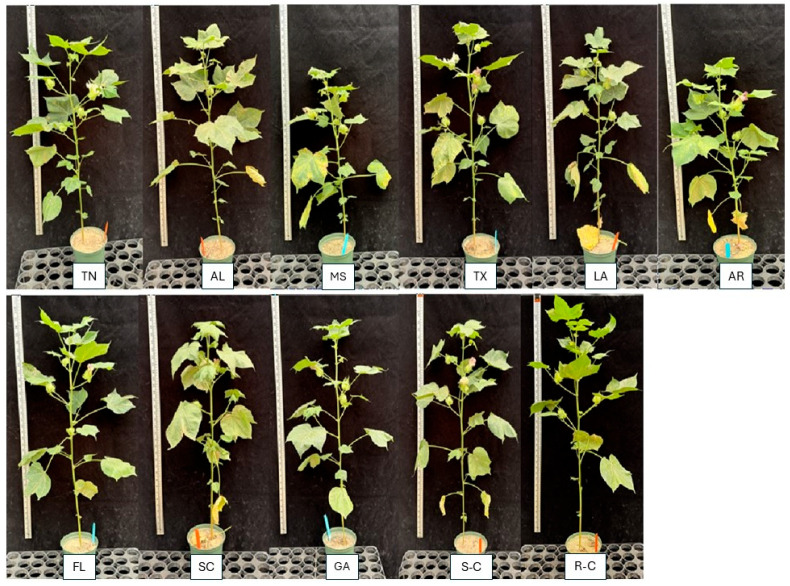
Height of cotton plants (DP 2141NR B3XF) at 60 days after inoculation compared with non-inoculated resistant (DP 2141NR B3XF) and susceptible (DP 2317 B3TXF) controls. Nematode isolates are designated as follows: TN = Tennessee, AL = Alabama, MS = Mississippi, TX = Texas, LA = Louisiana, AR = Arkansas, FL = Florida, SC = South Carolina, and GA = Georgia. S-C = susceptible control, R-C = resistant control.

Plant heights were impacted by the main and interactive effects of cotton cultivar and nematode isolate (Table 1). The heights were significantly reduced for the plants inoculated with any nematode isolate when compared with the non-inoculated control at 30 and 60 DAI. At 30 DAI, plant heights of nematode-inoculated plants remained statistically similar when compared among the isolates. Among all isolates at 60 DAI, plants inoculated with the AL isolate had statistically greater heights while the plants inoculated with the MS isolate remained at a statistically lower level (Figure 1). The resistant cultivar had significantly greater plant height relative to the susceptible cultivar, with the increment being 2% at 30 DAI and 3% at 60 DAI.

**Figure 2:**
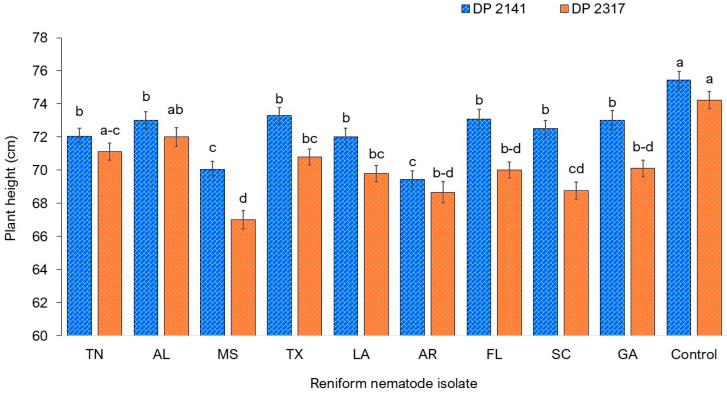
Interactive effect of reniform nematode isolate and cotton cultivar on height of the cotton plants at 60 days after inoculation. Plants were inoculated with 10,000 eggs per pot and were maintained in a greenhouse environment. Data were combined over two experiments and are means of ten replications. Treatment means followed by a common letter across the same color bars are not significantly different according to Tukey’s HSD test (*p* ≤ 0.05). Nematode isolates are designated as follows: TN = Tennessee, AL = Alabama, MS = Mississippi, TX = Texas, LA = Louisiana, AR = Arkansas, FL = Florida, SC = South Carolina, and GA = Georgia. DP 2141 = DP 2141NR B3XF (resistant), DP 2317 = DP 2317 B3TXF (susceptible).

The interactive effect of nematode isolate and cotton cultivar was observed at 60 DAI (Table 1 and Figure 2). The resistant cultivar inoculated with the MS and AR isolates had statistically the lowest plant height relative to the control, while the remaining isolates had intermediate effects between these two isolates (MS and AR) and the control. The susceptible cultivar inoculated with the MS isolate had the lowest plant height, which was statistically lower than the plants inoculated with TN, AL, TX, LA isolates and the control. The impact of TN and AL isolates on the susceptible cultivar was at the same statistical significance as the control.

### 3.2. Impact on Leaf

The origin of nematode isolate as well as cotton cultivar impacted the number of cotton leaves (Table 1). At 30 DAI, the number of leaves on the AL isolate-inoculated plants remained at the same statistical level as the control while the plants inoculated with other isolates had significantly less leaves. Plants inoculated with the MS, LA, AR, and SC isolates had significantly less leaves while the impact of the rest of the isolates remained similar to the control. The resistant cultivar produced a greater number of leaves relative to the susceptible cultivar at 30 DAI and 60 DAI. No interactive effect of nematode isolate and cotton cultivar was observed.

### 3.3. Impact on Cotton Boll

The origin of nematode isolate as well as cotton cultivar influenced the cotton boll weight, but it was not influenced by their interactive effects (Table 1). The AL, MS, TX, LA, AR, and FL isolates significantly impacted the cotton boll weight while the impact of other isolates on boll weight remained statistically similar to the control. The resistant cultivar had 79% greater boll weight than the susceptible cultivar.

### 3.4. Impact on Plant Vigor

Plant vigor was impacted by the main as well as interactive effects of nematode isolate and cotton cultivar (Table 2). Plants inoculated with the AR and SC isolates had significantly lower vigor than the control at 15 DAI, while the vigor of plants inoculated with other isolates was statistically similar to the control. At 30 DAI, all but the AL isolate-inoculated plants had significantly lower vigor than the control, with the AR isolate-inoculated plants being the least vigorous. Plants inoculated with any nematode isolate had significantly lower vigor at 45 DAI and 60 DAI compared to the control. Among the nematode-inoculated plants, the AR-inoculated plants were the least vigorous and the AL-inoculated plants were the most vigorous at 45 DAI. The AR-inoculated plants were the least vigorous among nematode-inoculated plants at 60 DAI. Relative to the resistant cultivar, the vigor of the susceptible cultivar was reduced by 12% at 15 DAI, by 3% at 30 DAI and 45 DAI, and by 5% at 60 DAI.

The interactive effect of nematode isolate and cotton cultivar on plant vigor was observed at 30, 45 and 60 DAI, but not at 15 DAI (Table 2). The resistant plants inoculated with any isolates remained more vigorous than the susceptible plants at 30, 45 and 60 DAI, with the AR-inoculated plants being the least vigorous (Table 3). While the NDVI values of nematode-inoculated plants differed significantly among the isolates for each cultivar at 30 DAI and 45 DAI, these values for the susceptible cultivar remained statistically similar among the isolates at 60 DAI (Table 3).

### 3.5. Impact on Chlorophyll Content

Chlorophyll content of cotton plants was influenced by the main and interactive effects of nematode isolate and cotton cultivar, although not always at significant levels (Table 4). At 15 DAI, plants inoculated with any of the nematode isolates had lower SPAD relative to the control. Chlorophyll content of the nematode-inoculated plants at 30 DAI and 60 DAI was also significantly lower than the control, with the AR isolate-inoculated plants having the least chlorophyll. At 15 and 30 DAI, the resistant cultivar had similar or statistically lower chlorophyll than the susceptible cultivar, while at 60 DAI it contained significantly higher chlorophyll.

**Figure 3:**
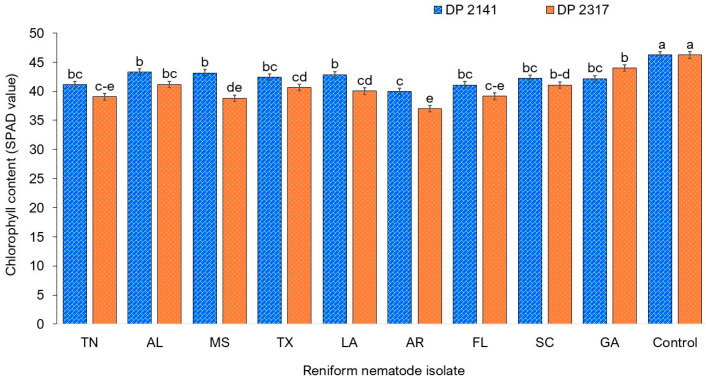
Interactive effect of reniform nematode isolate and cotton cultivar on leaf chlorophyll content at 60 days after inoculation. Plants were inoculated with 10,000 eggs per pot and were maintained in a greenhouse environment. Data were combined over two experiments and are means of ten replications. Treatment means followed by a common letter across the same color bars are not significantly different according to Tukey’s HSD test (*p* ≤ 0.05). Nematode isolates are designated as follows: TN = Tennessee, AL = Alabama, MS = Mississippi, TX = Texas, LA = Louisiana, AR = Arkansas, FL = Florida, SC = South Carolina, and GA = Georgia. SPAD = soil–plant analysis development, DP 2141 = DP 2141NR B3XF (resistant), DP 2317 = DP 2317 B3TXF (susceptible).

The interactive effect of nematode isolate and cotton cultivar on leaf chlorophyll content was observed only at 60 DAI (Figure 3 and Table 4). The AR isolate-inoculated resistant and susceptible plants had the least chlorophyll content relative to the controls, with the reduction being 14% and 20%, respectively. The AL isolate-inoculated resistant and susceptible plants had the least chlorophyll reduction, with the reductions being 6% and 11%, respectively, relative to the controls. The impacts of other isolates on the chlorophyll content of both the resistant and susceptible cultivars were intermediate.

### 3.6. Impact on Photosynthesis and Transpiration

Photosynthesis of cotton plants was not influenced by nematode isolate and cotton cultivar or by their interaction (Table 4). Transpiration was influenced by cotton cultivar, with the resistant cultivar exhibiting significantly greater transpiration. Nematode isolate as well as its interaction with cotton cultivar did not influence the transpiration of cotton.

### 3.7. Correlation Between Nematode Reproduction and Parameters of Cotton Growth and Physiology

**Figure 4:**
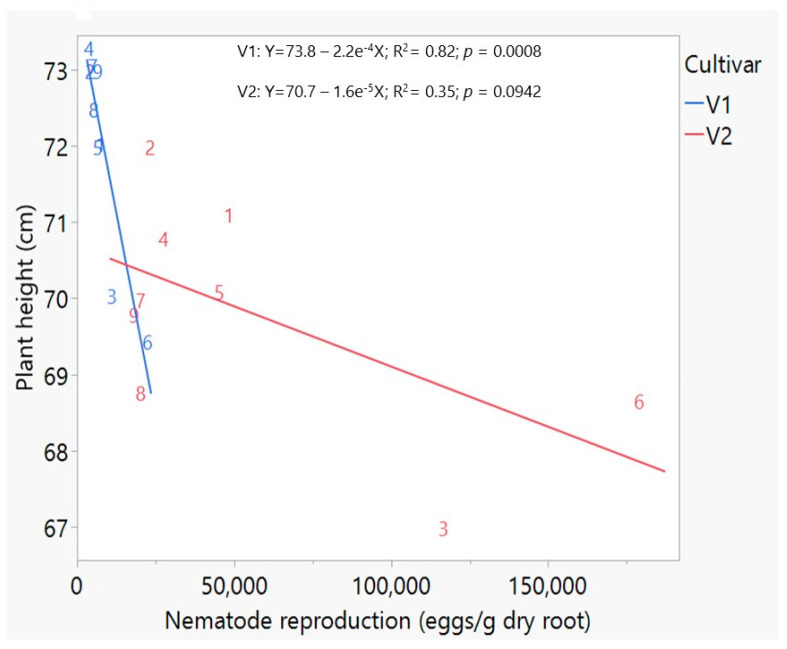
Linear relationship between reniform nematode reproduction and plant height for resistant (DP 2141 NR B3XF, V1) or susceptible (DP 2317 B3TXF, V2) cotton cultivars. Similar numbers in red or blue represent the same nematode isolate. Nematode isolates are designated as follows: 1 = Tennessee, 2 = Alabama, 3 = Mississippi, 4 = Texas, 5 = Louisiana, 6 = Arkansas, 7 = Florida, 8 = South Carolina, and 9 = Georgia.

**Figure 5:**
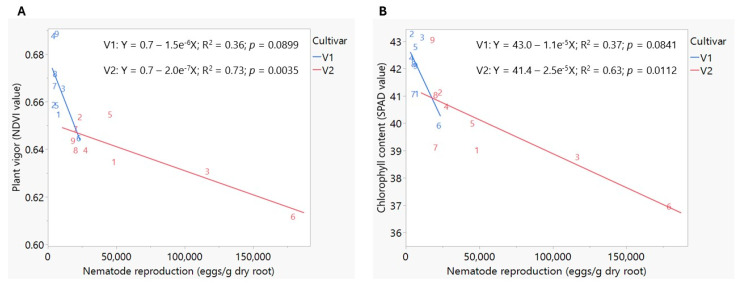
Linear relationship between nematode reproduction and plant vigor (**A**) and leaf chlorophyll content (**B**) for resistant (DP 2141 NR B3XF, V1) or susceptible (DP 2317 B3TXF, V2) cotton cultivars. Similar numbers in red or blue represent the same nematode isolate. Nematode isolates are designated as follows: 1 = Tennessee, 2 = Alabama, 3 = Mississippi, 4 = Texas, 5 = Louisiana, 6 = Arkansas, 7 = Florida, 8 = South Carolina, and 9 = Georgia.

The height and vigor of cotton plants as well as leaf chlorophyll content at the time of experiment termination were negatively impacted by nematode reproduction (Figure 2 and Figure 3). A significant correlation between nematode reproduction and plant height was observed for the resistant cultivar, but not for the susceptible cultivar (Figure 4). An 82% variation in plant height of the resistant cultivar was explained by nematode reproduction (Figure 4). The correlation between plant vigor as well as leaf chlorophyll content and nematode reproduction was significant for the susceptible cultivar but not for the resistant cultivar (Figure 5). Nematode reproduction explained 73% of variability in plant vigor of the susceptible cultivar (Figure 5A). Similarly, nematode reproduction explained 63% of leaf chlorophyll content variation in the susceptible cultivar (Figure 5B).

## 4. Discussion

The reniform nematode is one of the major constraints of cotton production. Management of this nematode continues to be a challenge for cotton producers due to the lack of safer fumigant alternatives that are as effective as methyl bromide. A new hope for reniform nematode management was ignited following the release of some reniform nematode-resistant cotton cultivars; however, field studies indicate a lack of uniform suppression of nematodes across wider geographic locations [8,9,10]. While the question remains concerning what drives the differential response of resistant cotton cultivars, the answer may lie in the variability in reniform nematode populations. Our recently published study found substantial differences in reproduction and virulence among reniform nematode populations of different geographic origin, implying one of the reasons behind the lack of uniform performance of resistant cotton cultivars is due to inherent variability in the nematode [15]. This study further examined the differential impacts of reniform nematodes originating from nine geographic locations on the development of resistant and susceptible cotton cultivars. While previous reports have mentioned reniform nematode adversely impacting cotton growth and yields [13,21], to the best of our knowledge, this study is the first to quantify the impact of reniform nematode on the development of cotton.

One of the most noticeable impacts of reniform nematode infection on crops is stunted growth. Reduction in yield becomes obvious when plants do not achieve desirable heights. Our study found that the height of cotton plants inoculated with any of the isolates was significantly reduced at both 30 DAI and 60 DAI relative to the control. These differences will likely be greater in field conditions where cotton plants are grown for a full season. As the impact of the reniform nematode on plant height varied greatly by their origin, understanding the aggressiveness of this nematode can help develop region-specific reniform nematode management strategies in cotton. Additionally, cotton plants of the resistant cultivar were taller than the susceptible cultivar at both 30 DAI and 60 DAI indicating the employment of resistant cultivars provides improved plant growth while also achieving nematode suppression.

Variation was observed on the ability of the different reniform nematode isolates to impact plant height as cotton continued to grow. This variation was pronounced at 60 DAI. Plants inoculated with the AR and MS isolates sustained the greatest losses in heights, implying greater impacts of these isolates. In our recently published study, we found these isolates were also the greater reproductive isolates [15], implying a positive correlation between nematode reproduction and plant growth in cotton. However, the relationship between nematode reproduction and plant damage may not always be positively correlated. A previous study reported the least amount of damage to soybeans by the most reproductive isolate [22]. Cotton plants inoculated with other isolates in this study had relatively greater heights, and these isolates were previously reported to be low reproductive ones [15], further implying a positive relationship between nematode reproduction and cotton growth. While resistant plants inoculated with any of the isolates were taller than the susceptible plants, only the MS- and the SC isolate-inoculated resistant plants experienced significant increase in plant height.

The differential ability of reniform nematode isolates to impact the number of leaves and boll weights was similar to the impact on plant height, implying a careful assessment of nematode biology is necessary for selecting a sustainable reniform nematode management strategy. Studies documenting the impact of reniform nematodes on the number of cotton leaves are lacking. A previous study conducted in a greenhouse, however, documented the negative impact of root-knot nematode on cotton boll weight [20]. Another study reported root-knot and lance nematodes negatively impacting leaf numbers of mulberry [23]. Reniform nematode has been reported to reduce cotton yield by 40% to 60% [19], and this estimate closely resembled a 42% to 66% loss in cotton boll weight in our study. This result indicates that a significant cotton yield loss to reniform nematode can be expected when susceptible cotton is planted. Provided our study was conducted for a short duration, reniform nematode damage in the entire crop-growing season under field conditions can go even higher than what we found under greenhouse conditions.

Changes in physiological parameters of plants are often assessed as a measure of crop development. This study found that cotton physiological parameters were differentially impacted by the origin of nematode isolates. Plants inoculated with the AL isolate had better vigor throughout the study period while the AR isolate-inoculated plants had the least vigor. Additionally, plant vigor readings were the lowest at 15 DAI and the greatest at 30 DAI, and these readings were slightly reduced at 45 DAI and 60 DAI, probably indicating initial plant stress following nematode attack and their subsequent attempt to develop thereafter. The reduction in vigor at 45 DAI and thereafter could have been the result of the plants reaching the later stages of vegetative growth and the onset of reproductive growth [24]. While some studies have reported a substantial reduction in the vigor of cotton upon reniform nematode infection [10], results from our study suggested that care should be taken while interpreting plant vigor data as increased vigor may not always be indicative of reduced nematode impact. The continuous increase in chlorophyll content reflected by the SPAD measurements throughout the study period implied continuous expansion and maturity of the cotton leaves despite nematode infection. However, the magnitude of increase in chlorophyll in plants differed among the plants inoculated with nematode isolates at 30 DAI and 60 DAI. The isolate with higher virulence in our previous study [15] had lower chlorophyll content, and vice versa, meaning highly virulent isolates exerted greater physiological stress while less virulent isolates exerted lower physiological stress to cotton. Although there are no prior studies showing how leaf chlorophyll content changes upon reniform nematode infection over time in cotton, a study reported a reduction in chlorophyll content of cotton in response to increasing levels of root-knot nematode inoculum [20]. The photosynthesis and transpiration of plants inoculated with reniform nematode in our study remained unaffected, indicating no direct correlation between these physiological parameters and nematode infection. However, this study was conducted in a controlled environment for two months, and results may differ when assessed for a full season in field environments. Although prior studies assessing the impact of reniform nematode on photosynthetic ability and transpiration of cotton are not available, a growth cabinet study from the 1970s reported a significant reduction in the photosynthesis of tomato plants as soon as a day after inoculation with root-knot nematode [25], while another study involving multiple trials did not find consistent associations between photosynthesis and transpiration of cotton plants and root-knot nematode infection [20]. Because changes in crop physiology due to nematode infection likely impact cotton yield and fiber quality [20], multilocation field studies are needed to understand the relationship between reniform nematode infection and cotton physiology.

The resistant cotton cultivar upon reniform nematode infection having significantly greater plant height, number of leaves, dry boll weight, plant vigor, leaf chlorophyll content and transpiration relative to the susceptible cultivar indicated the greater yield potential of resistant cultivars is due to their improved physiological parameters, in addition to their ability to suppress nematode reproduction and virulence as reported in our previous study [15]. While the mechanism of resistance is still unknown to the public, resistance was likely derived from three genes *Ren^barb1^*, *Ren^barb2^*, and *Ren^barb3^* of *G. barbadense* accession GB 713 [26,27]. The regression analysis in our study found plant vigor and chlorophyll content of resistant cultivars were less affected by nematode reproduction (egg production on roots) in comparison with the susceptible cultivar, implying their better performance across nematodes of different origins. As cotton industries are continuously improving reniform nematode resistance through their breeding programs, employment of resistant cultivars will likely serve as a sustainable tool for reniform nematode management while improving crop yield.

Evolutionary forces such as mutation, genetic drift, migration, and natural selection drive diversity in nematodes [28,29]. These diversities in turn lead to adaptability to their existing environment through dynamics in life cycle, host exploitation, and efficient reproduction strategies [29,30]. Such evolutionary differences might have contributed to the differential ability of the reniform nematode isolates to impact cotton development in our study. Cotton plants inoculated with reniform nematode originating from the delta regions (AR, MS, TN) in our study exhibited greater adverse impacts on growth and physiological parameters, implying possible local adaptation or shared evolutionary history among these geographically proximate populations. These isolates also exhibited superiority in reproduction and virulence [15], which is an indication that geographical proximity can have profound effects on the ability of nematodes to reproduce and impact crop development. Conversely, this also implies that biologically different reniform nematode populations may not be managed using the same management strategy. Therefore, understanding the isolate-specific impact on a host plant can provide a foundation for the sustainable management of reniform nematode. Deployment of niche-specific nematode management strategies informed by nematode biology, such as careful selection of cultivars, nematicides, and rotation crops, may help reduce selection pressure on the nematode thereby contributing to sustainable nematode management. Understanding inherent genetic differences among geographically distinct populations of reniform nematode would aid in the development of biology-informed niche-specific management methods.

This study provides a quantitative assessment of the changes in cotton development (growth and physiology) in response to infection by the reniform nematode. Additionally, this study elucidated that cotton responds differently to nematode infection depending on the origin of the nematodes and that the response differs between resistant and susceptible cultivars. While the current study analyzed only a few parameters of cotton, assessing other parameters such as root architectural changes, stomatal conductance, water-use efficiency, nutrient uptake efficiency, lint yield, and lint quality would help make better decisions on reniform nematode management. Additional studies involving geographically more diverse populations are needed to further understand the response of cotton to geographically distinct populations of reniform nematode.

## Figures and Tables

**Table 1:** Main and interactive effects of reniform nematode isolate and cotton cultivar on plant height, number of leaves, and boll weight.

Factor	Treatment Level	Plant Height (cm)		Number of Leaves		Dry Boll Weight (g)
		30 DAI	60 DAI	30 DAI	60 DAI	
Cultivar (C)	DP 2141	52.65 ± 0.31 a	72.40 ± 0.17 a	19 ± 0.15 a	29 ± 0.33 a	4.11 ± 0.22 a
	DP 2317	51.40 ± 0.33 b	70.24 ± 0.17 b	18 ± 0.16 b	27 ± 0.34 b	2.30 ± 0.23 b
Isolate (I)	Tennessee	51.67 ± 0.72 b	71.58 ± 0.36 bc	18 ± 0.34 b	28 ± 0.73 ab	3.06 ± 0.50 ab
	Alabama	52.58 ± 0.72 b	72.50 ± 0.38 b	19 ± 0.34 ab	29 ± 0.73 ab	3.68 ± 0.50 b
	Mississippi	50.89 ± 0.72 b	68.52 ± 0.37 e	18 ± 0.39 b	26 ± 0.77 b	3.00 ± 0.56 b
	Texas	51.57 ± 0.70 b	72.05 ± 0.35 bc	18 ± 0.34 b	28 ± 0.73 ab	3.22 ± 0.50 b
	Louisiana	51.75 ± 0.72 b	71.05 ± 0.36 bc	18 ± 0.34 b	27 ± 0.75 b	3.41 ± 0.50 b
	Arkansas	50.64 ± 0.77 b	69.05 ± 0.41 de	18 ± 0.35 b	28 ± 0.75 b	2.18 ± 0.53 b
	Florida	51.35 ± 0.70 b	71.53 ± 0.38 bc	18 ± 0.35 b	29 ± 0.73 ab	3.38 ± 0.49 b
	South Carolina	51.76 ± 0.72 b	70.63 ± 0.36 cd	18 ± 0.38 b	28 ± 0.75 b	3.62 ± 0.48 ab
	Georgia	51.65 ± 0.70 b	71.40 ± 0.38 bc	18 ± 0.35 b	29 ± 0.73 ab	3.55 ± 0.50 ab
	Control	56.40 ± 0.70 a	74.83 ± 0.37 a	20 ± 0.34 a	31 ± 0.73 a	6.35 ± 0.48 a
*p*-value	C	0.0065	<0.0001	<0.0001	<0.0001	0.0006
	I	<0.0001	<0.0001	<0.0001	<0.0003	<0.0001
	C × I	0.9999	0.0295	0.7532	0.9666	0.9743

Plants were inoculated with 10,000 eggs per pot and were maintained in a greenhouse environment. Data were combined over two experiments and are means of ten replications. Analysis of variance was conducted using Student’s *t*-test or Tukey’s HSD (*p* ≤ 0.05). Treatment means are presented as mean ± standard error and are not significantly different when followed by a common letter within a column. Cotton bolls after recording fresh boll weight were dried at 65 °C for a week to record the dry boll weight. DAI = days after inoculation, DP 2141 = DP 2141 NR B3XF (resistant), DP 2317 = DP 2317 B3TXF (susceptible).

**Table 2:** Main and interactive effects of reniform nematode isolate and cotton cultivar on plant vigor.

Factor	Treatment Level	Plant Vigor (NDVI Value)			
		15 DAI	30 DAI	45 DAI	60 DAI
Cultivar (C)	DP 2141	0.27 ± 0.003 a	0.73 ± 0.003 a	0.63 ± 0.002 a	0.67 ± 0.002 a
	DP 2317	0.24 ± 0.003 b	0.71 ± 0.003 b	0.61 ± 0.002 b	0.64 ± 0.002 b
Isolate (I)	Tennessee	0.27 ± 0.007 a–c	0.71 ± 0.007 b–d	0.61 ± 0.005 b–e	0.64 ± 0.005 bc
	Alabama	0.27 ± 0.007 ab	0.74 ± 0.006 ab	0.63 ± 0.005 b	0.65 ± 0.005 b
	Mississippi	0.24 ± 0.007 b–d	0.70 ± 0.006 cd	0.60 ± 0.005 c–e	0.64 ± 0.005 bc
	Texas	0.25 ± 0.007 a–d	0.72 ± 0.007 bc	0.62 ± 0.005 bc	0.66 ± 0.005 b
	Louisiana	0.26 ± 0.007 a–c	0.72 ± 0.007 bc	0.62 ± 0.005 bc	0.65 ± 0.006 b
	Arkansas	0.22 ± 0.007 d	0.69 ± 0.006 d	0.59 ± 0.005 e	0.62 ± 0.005 c
	Florida	0.28 ± 0.007 a	0.71 ± 0.006 b–d	0.60 ± 0.005 de	0.65 ± 0.006 b
	South Carolina	0.23 ± 0.007 cd	0.72 ± 0.007 bc	0.61 ± 0.005 b–e	0.65 ± 0.005 b
	Georgia	0.24 ± 0.007 b–d	0.72 ± 0.007 bc	0.62 ± 0.005 b–d	0.66 ± 0.005 b
	Control	0.27 ± 0.007 ab	0.76 ± 0.006 a	0.70 ± 0.005 a	0.71 ± 0.005 a
*p*-value	C	<0.0001	<0.0001	<0.0001	<0.0001
	I	<0.0001	<0.0001	0.0013	<0.0001
	C × I	0.0719	0.0123	<0.0001	0.0416

Plants were inoculated with 10,000 eggs per pot and were maintained in a greenhouse environment. Data were combined over two experiments and are means of ten replications. Analysis of variance was conducted using Student’s *t*-test or Tukey’s HSD (*p* ≤ 0.05). Treatment means are presented as mean ± standard error and are not significantly different when followed by a common letter within a column. NDVI values at 45 DAI were transformed using the square root function of JMP PRO 18.0. NDVI = normalized digital vegetation index, DAI = days after inoculation, DP 2141 = DP 2141 NR B3XF (resistant), DP 2317 = DP 2317 B3TXF (susceptible).

**Table 3:** Interactive effect of reniform nematode isolate and cotton cultivar on plant vigor.

	Plant Vigor (NDVI Value)					
Isolate	30 DAI		45 DAI		60 DAI	
	Cultivar		Cultivar		Cultivar	
	DP 2141	DP 2317	DP 2141	DP 2317	DP 2141	DP 2317
Tennessee	0.73 ± 0.009 ab	0.68 ± 0.009 c–e	0.61 ± 0.007 bc	0.60 ± 0.007 b–d	0.65 ± 0.008 c	0.63 ± 0.008 b
Alabama	0.74 ± 0.009 ab	0.73 ± 0.009 ab	0.63 ± 0.007 bc	0.63 ± 0.007 b	0.65 ± 0.008 bc	0.65 ± 0.008 b
Mississippi	0.72 ± 0.009 b	0.68 ± 0.009 de	0.62 ± 0.007 bc	0.58 ± 0.007 d	0.66 ± 0.008 bc	0.63 ± 0.008 b
Texas	0.72 ± 0.009 b	0.73 ± 0.009 a–c	0.65 ± 0.007 b	0.60 ± 0.007 b–d	0.68 ± 0.008 ab	0.64 ± 0.008 b
Louisiana	0.72 ± 0.009 b	0.72 ± 0.009 a–e	0.65 ± 0.007 b	0.60 ± 0.008 b–d	0.65 ± 0.008 bc	0.65 ± 0.008 b
Arkansas	0.70 ± 0.009 b	0.67 ± 0.009 e	0.59 ± 0.007 c	0.58 ± 0.007 cd	0.64 ± 0.008 c	0.61 ± 0.008 b
Florida	0.72 ± 0.009 b	0.69 ± 0.009 b–e	0.60 ± 0.007 c	0.59 ± 0.007 b–d	0.66 ± 0.008 bc	0.65 ± 0.009 b
South Carolina	0.73 ± 0.009 ab	0.70 ± 0.009 b–e	0.63 ± 0.007 bc	0.60 ± 0.007 b–d	0.67 ± 0.008 bc	0.64 ± 0.008 b
Georgia	0.72 ± 0.009 b	0.72 ± 0.009 a–d	0.62 ± 0.007 bc	0.61 ± 0.007 bc	0.68 ± 0.008 ab	0.64 ± 0.008 b
Control	0.77 ± 0.009 a	0.76 ± 0.009 a	0.71 ± 0.007 a	0.71 ± 0.007 a	0.71 ± 0.008 a	0.71 ± 0.008 a

Plants were inoculated with 10,000 eggs per pot and were maintained in a greenhouse environment. Data were combined over two experiments and are means of ten replications. Analysis of variance was conducted using Student’s *t*-test or Tukey’s HSD (*p* ≤ 0.05). Treatment means are presented as mean ± standard error and are not significantly different when followed by a common letter within a column. NDVI values at 45 DAI were transformed using the square root function of JMP PRO 18.0. NDVI = normalized digital vegetation index, DAI = days after inoculation, DP 2141 = DP 2141 NR B3XF (resistant), DP 2317 = DP 2317 B3TXF (susceptible).

**Table 4:** Main and interactive effects of reniform nematode isolate and cotton cultivar on leaf chlorophyll content, photosynthesis, and transpiration of cotton plants.

		Chlorophyll Content (SPAD Value)			Photosynthesis (µ mol CO_2_/m^2^s)	Transpiration (µ mol H_2_O/m^2^s)
Factor	Treatment Level	15 DAI	30 DAI	60 DAI		
Cultivar (C)	DP 2141	34.83 ± 0.28 b	36.40 ± 0.15 a	42.44 ± 0.18 a	12.00 ± 0.37 a	5.23 ± 0.16 a
	DP 2317	35.80 ± 0.29 a	36.16 ± 0.15 a	40.61 ± 0.18 b	10.97 ± 0.37 a	4.65 ± 0.16 b
Isolate (I)	Tennessee	35.45 ± 0.63 b	36.51 ± 0.32 bc	40.07 ± 0.40 cd	11.13 ± 0.82 a	4.94 ± 0.35 a
	Alabama	35.38 ± 0.65 b	36.21 ± 0.32 bc	42.22 ± 0.39 b	12.24 ± 0.82 a	5.21 ± 0.35 a
	Mississippi	35.66 ± 0.63 b	34.57 ± 0.33 d	40.97 ± 0.40 bc	10.33 ± 0.82 a	4.67 ± 0.35 a
	Texas	35.34 ± 0.63 b	36.10 ± 0.32 bc	41.53 ± 0.39 bc	11.71 ± 0.82 a	5.06 ± 0.35 a
	Louisiana	34.33 ± 0.63 b	37.13 ± 0.32 b	41.42 ± 0.39 bc	11.91 ± 0.82 a	5.24 ± 0.35 a
	Arkansas	34.91 ± 0.63 b	34.34 ± 0.32 d	38.46 ± 0.39 d	9.52 ± 0.82 a	4.25 ± 0.35 a
	Florida	34.73 ± 0.63 b	35.34 ± 0.32 cd	40.11 ± 0.40 cd	11.21 ± 0.82 a	4.87 ± 0.35 a
	South Carolina	34.42 ± 0.63 b	36.60 ± 0.32 bc	41.62 ± 0.39 bc	11.62 ± 0.82 a	5.10 ± 0.35 a
	Georgia	34.24 ± 0.63 b	36.26 ± 0.32 bc	42.60 ± 0.39 b	11.52 ± 0.82 a	4.77 ± 0.35 a
	Control	38.63 ± 0.67 a	39.68 ± 0.33 a	46.24 ± 0.40 a	13.67 ± 0.82 a	5.25 ± 0.35 a
*p*-Value	C	0.0186	0.2418	<0.0001	0.0500	0.0105
	I	0.0003	<0.0001	0.0005	0.0752	0.6318
	C × I	0.3361	0.4507	<0.0001	0.9981	0.4533

Plants were inoculated with 10,000 eggs per pot and were maintained in a greenhouse environment. Data were combined over two experiments and are means of ten replications. Analysis of variance was conducted using Student’s *t*-test or Tukey’s HSD (*p* ≤ 0.05). Treatment means are presented as mean ± standard error and are not significantly different when followed by a common letter within a column. Plant vigor data at 45 DAI were transformed using the square root function of JMP PRO 18.0. DAI = days after inoculation, SPAD = soil–plant analysis development, DP 2141 = DP 2141 NR B3XF (resistant), DP 2317 = DP 2317 B3TXF (susceptible).

## Data Availability

The original contributions presented in this study are included in the article. Further inquiries can be directed to the corresponding author.
